# Combinatorial Antimicrobial Effects of Imidazolium-Based Ionic Liquids and Antifungals on Model Fungal Organisms

**DOI:** 10.3390/biom15121657

**Published:** 2025-11-27

**Authors:** Jesus G. Calixto, Peter R. Fetz, Daniel Ammerman, Yesenia R. Flores, Gregory A. Caputo, Timothy D. Vaden, Benjamin R. Carone

**Affiliations:** 1 Department of Biology & Biomedical Sciences, Rowan University, 201 Mullica Hill Rd, Glassboro, NJ 08028, USA; jec398@pitt.edu (J.G.C.); p.remsen.fetz@gmail.com (P.R.F.);; 2 Department of Chemistry & Biochemistry, Rowan University, 201 Mullica Hill Rd, Glassboro, NJ 08028, USAvadent@rowan.edu (T.D.V.)

**Keywords:** antifungal, ionic liquid, combinatorial treatment

## Abstract

Ionic Liquids (IL) are a unique class of molten salts, with specific formulations exhibiting antimicrobial properties. Several recent studies have highlighted the ability of ILs to form micelles, permeabilize the plasma membrane, and destabilize cellular structure, ultimately initiating cell death. Moreover, while these membrane-destabilizing properties are cytotoxic to most cellular organisms at high concentrations, their membrane destabilization capability at lower concentrations may lead to improvements in drug delivery for combinatorial therapies against specific microbes. Work presented in this study aimed to identify a synergistic relationship between ILs, 1-n-Hexyl-3-methylimidazolium chloride (HMIM[Cl]) and 1-Methyl-3-n-octylimidazolium chloride (OMIM[Cl]), and antifungal drugs (AF), Clotrimazole, Ketoconazole, Fluconazole, and Itraconazole, with the hypothesis that in a combinatory setting there should be improved AF efficacy against model fungal organisms: *S. boulardii*, *S. cerevisiae*, *S. pombe*, and *C. albicans*. Several complementary assays were used to identify the combined effects of IL + AF treatment, including Kirby–Bauer tests and minimum inhibitory concentrations (MIC) assays to establish antimicrobial effects, and flow cytometry to evaluate cell wall permeability. Finally, we demonstrate that at low concentrations, the ILs tested in this study are capable of improving the effectiveness of current antifungal compounds at concentrations not cytotoxic to human cells.

## 1. Introduction

Fungal pathogens pose a major health challenge, contributing to over 1.6 million deaths annually, with *Candida* species (spp.) as the worldwide leading pathogens [1]. In healthy individuals, *Candida* spp. are most often commensal and frequently found in the human body, namely, on the surface of skin, and in the gastrointestinal, respiratory, and genitourinary tracts. However, in immunocompromised or ill individuals, *Candida* spp. can become opportunistic pathogens, causing infections that range from superficial and easily treatable, such as oral or vaginal, to severe systemic infections that can be life-threatening. Of these systemic infections, approximately 50–70% can be attributed to *Candida* spp. [2], with *C. albicans* being the most common species detected. Moreover, these infections are increasingly acquired in clinical settings and from medical devices, including catheters, heart valves, grafts, and tracheal tubes, which can incur high mortality rates [3,4]. Despite the long-standing use of multiple antifungal drug treatments, the efficacy of these existing treatments and development of new antifungals significantly lags the advances in antibacterials. Moreover, the increasing incidence of *Candida* spp. with resistance to the most used antifungals and other formerly reliable therapeutics, hallmarks the dire need for advancements in current human mycoses care.

Current treatments for candidiasis rely on four main antifungal drugs categorized as azoles, polyenes, allylamines, and echinocandins [5]. For example, one of the most used antifungals, Fluconazole, is widely used because of its affordability, high bioavailability, and flexible administration methods [6]. However, increasing resistance among *Candida* spp. has diminished its effectiveness in current therapeutic regimens [7]. Resistance mechanisms include drug efflux, alterations to target sites, and biofilm formation, which significantly enhances drug resistance [8]. Existing treatments are largely ineffective against biofilms, highlighting the urgent need for new antifungal agents that can disrupt biofilms or enhance drug delivery [9]. Identifying new ways to increase the effectiveness of existing antifungals and combat drug resistance in *Candida* spp. pathogens remains an important priority in developing new effective treatments.

One strategy to combat antimicrobial resistance and improve antimicrobial efficacy involves combining existing antimicrobial compounds with secondary compounds that enhance their performance [10,11]. This improvement can occur through two types of interactions: (1) combinatorial interactions, where the antimicrobial activity of the primary compound is improved by the addition of a secondary compound, and (2) synergistic interactions, where the combined effect of the antimicrobial and secondary compound exceeds the sum of their individual effects. In clinical practice, the use of combinatorial antimicrobial approaches has only been applied to a limited extent. One of the most widely used and best examples of combinatorial drug treatment in the clinic is amoxicillin combined with clavulanic acid. In this combination, amoxicillin inhibits peptidoglycan synthesis, while clavulanic acid blocks the β-lactamase enzyme that degrades amoxicillin [12]. Similar strategies involve combining two compounds that each exhibit independent antimicrobial activity, allowing for combined dosing during treatment [13]. In laboratory settings, promising results have been observed with combinations that enhance antimicrobial performance. Notably, synergistic interactions have been demonstrated between the antibiotic tetracycline and several secondary compounds, including 2-aminoimidazoles, quercetin, and baicalin [14,15,16]. Identifying novel molecules that can be successfully used in combined therapies is a rapidly growing field.

One potential candidate for combinatorial treatment approaches with antimicrobials is a class of molecules called Room Temperature Ionic Liquids (RTILs), which are salts that remain in a liquid state at room temperature without the need for a solvent [17]. RTILs belong to a specialized group of Ionic Liquids (ILs) that have recently garnered significant interest due to their broad range of potential applications. Generally, ILs consist of an anion-cation pair that can be combined in various ways, leading to a vast number of possible IL species. These compounds often display unique chemical properties, including low melting points, negligible vapor pressure, and exceptional solvation capabilities. Additionally, ionic liquids have attracted attention as tunable biomaterials—through modification of their molecular cations and anions—for diverse biomedical applications [18,19,20]. Studies have shown that ILs can stabilize or destabilize protein structures, enhance enzymatic activity, affect DNA conformation, and influence sensitive protein interactions [21,22,23]. Recently, ILs have been identified as candidates for combinatorial antimicrobial treatments. Studies in bacteria indicate that imidazolium-chloride-based ILs can act synergistically with a wide range of antibacterial drugs on a diverse set of bacteria spp. [24]. Additionally, these ILs have demonstrated standalone antimicrobial and antibiofilm activity against *C. albicans* [25] and yield low toxicity in human cell lines [26]. Whether ILs can act in combination with antifungals to enhance antimicrobial effects remains poorly studied, and the identification of ILs which enhance AF efficacy will lead to IL + AF formulations that may be immediately clinically relevant for non-invasive fungal infections.

The work presented herein investigates the combination of three specific imidazolium-based RTILs that contain variable-length alkyl chains with a panel of four traditional antifungal drugs to investigate the potential for combined enhanced antifungal efficacy. The data presented below evaluates a subset of well-studied RTILs, 1-alkyl-3-methylimidazolium, which are all liquid at room temperature. Finally, the combinations of these RTILs + antifungal compounds were screened for their additive antifungal effects and demonstrated enhanced antifungal activity in combinatorial applications.

## 2. Materials and Methods

### 2.1. Ionic Liquids (IL)

Ionic liquids used in this study are as follows: 1-ethyl-3-methylimidazolium chloride, [EMIM]Cl, Sigma; 1-hexyl-3-methylimidazolium chloride, [HMIM]Cl, Alfa Aesar; and 1-octyl-3-methylimidazolium chloride, [OMIM]Cl, Alfa Aesar. Formulations of IL at indicated concentrations were made with either water or PBS (Phosphate Buffer Saline as indicated by experiment.

### 2.2. Minimal Inhibitory Concentration (MIC)

Fungi were plated from frozen glycerol stocks (−80 °C) onto YPD agar plates: *S. boulardii* CNCM I-745, *S. cerevisiae* [27], *S. pombe* [28], and *C. albicans ATCC MYA-2876*. A single colony was picked from each streak to prepare overnight in 5 mL fresh YPD broth and placed into a tilted rotating incubator set at 30 °C and 40 rpm overnight. After ~16–20 h, a fresh dilution in media (1:200) was made and used for the experiment. Minimum Inhibitory Concentration (MIC) experiments were performed with mid-log phase yeast which was diluted to 5 × 10^3^ CFU/mL. Finally, diluted culture was added to each well of a sterile 96-well plate containing serially diluted aliquots of each RTIL to a total volume of 100 µL. The plate was incubated at 30 °C overnight with shaking for 24 and 48 h. At both timepoints, the yeast was manually resuspended in the plate by pipetting and we measured the OD_600_ using a Spectramax M5 multimode plate reader (Molecular Devices, San Jose, CA, USA).

### 2.3. Kirby–Bauer Disk Assays

Assessment of fungal growth on YPD plates with AFs + ILs served as an indicator for the combined efficacy of treatment of ILs and AFs. YPD agar plates were made with addition of increasing concentrations of [HMIM]Cl at 0, 2.5, 6.25, and 25 mM or [OMIM]Cl at 0, 0.25, 0.625, and 2.5 mM. Plates solidified and dried on bench for 24 h before use. IN total, 400 μL of *S. cerevisiae* or *C. albicans* liquid culture at mid-log phase OD_600_ 0.5 was spread using sterile glass beads to achieve even distribution. Following addition of culture, the plates were allowed to dry for 30 min before the addition of antifungal disks. Antifungal-infused disks were prepared using the stock solutions of solubilized antibiotics; 15 μL of each of the 4 azole antifungals at stock concentrations was pipetted onto a sterile 6 mm cellulose disk (BD). Stock concentrations of antifungals used in this study were prepared in DMSO near their maximum solubility: Fluconazole (100 mg/mL), Ketoconazole (20 mg/mL), Clotrimazole (20 mg/mL), and Itraconazole (10 mg/mL). After plate drying was complete, sterile forceps were used to transfer the disks to the media, and the plate was grown upright in an incubator overnight at 30 °C. The following day, photographs were taken of the plates to visualize the zone of inhibition (ZOI) created by the combined treatment. Adobe Illustrator 2023 CC was used to perform digital measurements of ZOI diameters.

### 2.4. Checkerboard Assays

*C. albicans* was grown overnight in YPD liquid media at 30 °C, at which point it was diluted into 10 mL of YPD media at 0.1 OD_600_ and was grown to mid-log phase 0.5 OD_600_ and subsequently diluted to 5 × 10^3^ CFU/mL cells in YPD of which 180 μL of culture was distributed into a 96-well round-bottom culture dish. To this dish, 20 μL of IL, AF, or IL + AF was added to each well at concentrations indicated in each experiment. These culture plates were grown in an incubator shaker for 48 h at 30 °C, at which point the cultures were mixed by pipetting and then spotted onto YPD solid plates and allowed to grow for an additional 48 h. After 48 h, plates were visually scored for no growth (0), 50% growth (1), or 100% growth relative to PBS control (2). Experiments were performed in triplicate, and scores were averaged per well.

### 2.5. Permeability Assay Flow Cytometry

*C. albicans* was grown to mid-log phase 0.5 OD_600_, and 180 μL of the culture was added to 96-well round-bottom culture plate. At time = 0, 20 μL IL, AF, or IL + AF was added to the plate and mixed by pipetting. These culture plates were then grown in an incubator shaker for 24 h at 30 °C. After 24 h, propidium iodide (PI) was added to each well to a final concentration of 5 mg/mL and incubated at 30 °C. Concentrations of IL, AF, and IL + AF were the same as those used for checkerboard assay. Cetyl-trimethyl-ammonium-bromide (CTAB) was used as a positive control at a final concentration of 0.3 M. Finally, internal fluorescence within cells was measured using a BD FACSCelesta instrument (BD Bioscience, San Deigo, CA, USA) in the PE-594 channel. At least 10,000 cells per well were counted for each treatment plate.

$$\frac{Ionic\;Liquid\;Sample}{CTAB\;Sample}=Degree\;of\;Permeabilization.$$

### 2.6. Treatment with HeLa Cells with ILs + Antifungals

HeLa cells (Kyoto line) [29] were grown at 37 °C in 5% CO_2_ using DMEM media with 4.5 g/L glucose + Sodium Pyruvate + L-Glutamine (VWRV02-0101) with 10% FBS. Antibiotics such as Pen/Strep were not used during treatment with ILs or AFs to exclude the possibility of interaction with other antibiotics. HeLa cells were grown to 70–90% confluency in T75 dishes; cells were dissociated with Trypsin/EDTA 0.125 M for 3 min and resuspended in 25 mL DMEM +10% FBS; 180 μL cells in media were added to each of the 96 wells on the plasma-treated culture plate and incubated at 37 °C overnight. Solutions of Clotrimazole plus either [HMIM]Cl and [OMIM]Cl were created and 20 μL of IL, AF, or IL + AF were added to 96-well plate and incubated at 37 °C for 24 h. Promega CellTiter-Blue Cell Viability reagent was used to evaluate cytotoxicity in accordance with the manufacturer’s guidelines. For each well, 20 μL of CellTiter-Blue was added and then the plates were incubated for either 1, 2, or 4 h and fluorescence was measured at Ex/EM 485/590 nm using a Biotek Synergy HT fluorometer Winooski, VT USA. EC50 and FIC calculations were performed using these treatments. EC50 calculations were performed using ATTB Bioquest online EC50 calculator and FIC scores were calculated as described in Doern et al. [30].

## 3. Results

### 3.1. Antimicrobial Activity of ILs and Antifungals in Yeast

**Figure 1:**
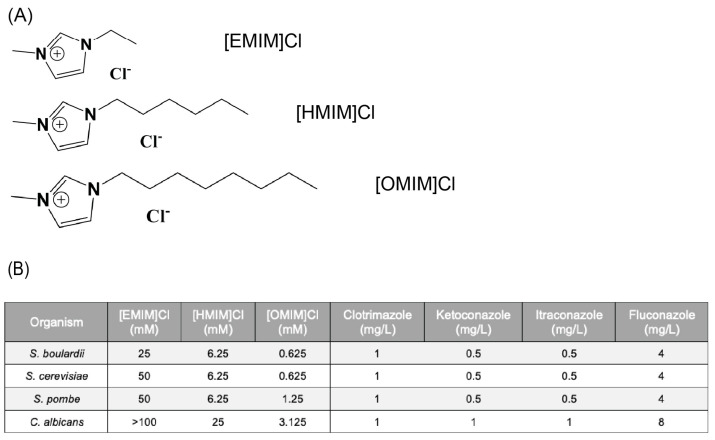
(**A**) Chemical structure of the three different ILs with differing alkyl chain lengths. (**B**) The minimum inhibitory concentration (MIC50) of the antifungals Clotrimazole, Ketoconazole, Itraconazole, and Fluconazole, along with Ionic Liquids (ILs) containing 1-alkyl-3-methylimidazolium and various alkyl chain lengths was determined on the yeast strains *S. boulardii*, *S. cerevisiae*, *S. pombe*, and *C. albicans*.

Previous studies have demonstrated a strong correlation between increased IL alkyl-chain length and minimal inhibitory concentration (MIC) in bacterial species [24,31]. To validate these results and establish baseline toxicity concentrations for yeast in this study, MIC50 concentrations were identified for three 3-methylimidazolium chloride ILs differing in alkyl chain length (Figure 1A) and four commonly used azole AFs in 4 yeast species. While many different imidazolium ILs with differing alkyl chain lengths are possible, previous work in bacteria suggests that chain lengths longer than OMIM[Cl] are cytotoxic and those shorter than BMIM[Cl] have no antimicrobial effect [24]. The four species of yeast tested included three common lab strains of non-pathogenic yeast strains, *S. boulardii*, *S. cerevisiae*, and *S. pombe*, and one common human pathogen, *C. albicans*. Using microbroth dilution methods adapted from the CSLI reference manual [32], MIC50 concentrations were calculated from OD_600_ nm values, indicating 50% growth after 48 h in YPD media. *C. albicans* was able to grow at higher concentrations of all three ILs tested and three of the four AFs, while all three other yeast strains evaluated were more sensitive to both ILs and AFs (Figure 1B). MIC50 values for *C. albicans* for the four tested AFs fell within the CSLI breakpoint values for these MIC50 values for non-resistant strains. Consistent with prior studies in bacteria, [EMIM]Cl exhibited very little cytotoxic effects and only inhibited fungal growth at very high concentrations [33]. Taken together, these results reaffirm results from past studies and suggest a more general trend correlating increased antimicrobial effects which increase with alkyl chain length.

### 3.2. Combination of ILs and Antifungals in Kirby–Bauer Disk Assays

**Figure 2:**
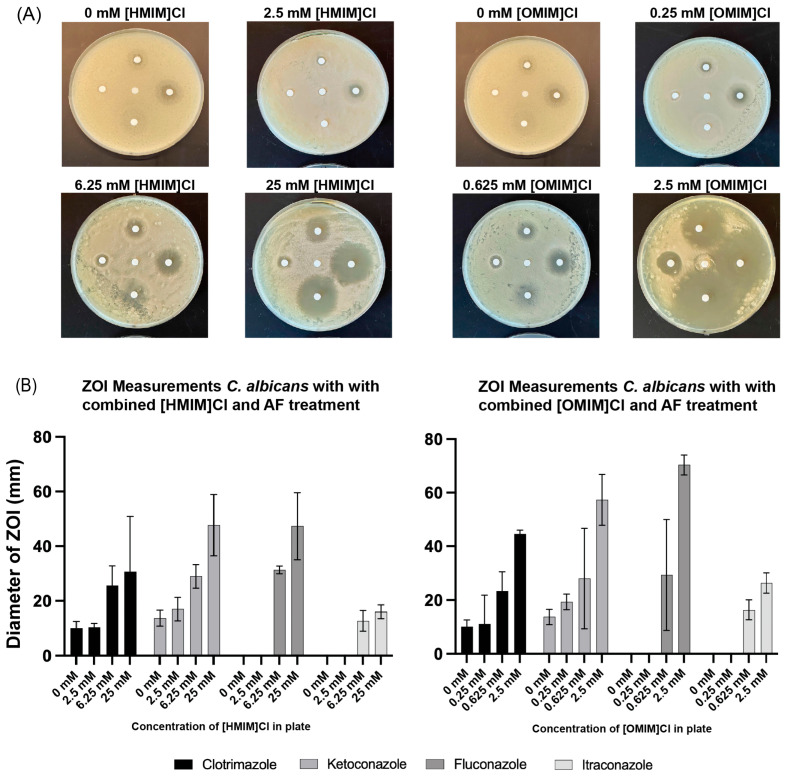
(**A**) Kirby-Bauer disk diffusion assay with indicated concentrations of IL in YPD media plates. Disk orientation from top in clockwise direction: Clotrimazole, Ketoconazole, Fluconazole, and Itraconazole. (**B**) Average diameter of Zones of Inhibition (ZOI) for Kirby–Bauer disk diffusion assay. Growth was performed in triplicate; error bars represent confidence intervals *p* < 0.05.

To assess the potential for combinatorial effects of ILs and AFs in yeast, Kirby–Bauer disk diffusion assays were performed for *C. albicans* and *S. cerevisiae* for [HMIM]Cl and [OMIM]Cl with all four AFs. YPD plates (120 mm) were created with the addition of [HMIM]Cl or [OMIM]Cl at concentrations around the MIC50 values for these compounds: [HMIM]Cl (0, 2.5, 6.25, or 25 mM) and [OMIM]Cl (0, 0.25, 0.625, or 2.5 mM). The addition of 6 mm cellulose disks infused with equal volumes of solubilized AFs to the plates occurred after plating of *C. albicans* or *S. cerevisiae*, allowing for growth for 48 h at 23 °C, at which point the plates were evaluated for the establishment of zones of inhibition (ZOI). Digital pictures were acquired of all plates, and measurements of ZOI diameter were performed digitally as described in the Methods. The negative control (PBS) demonstrated no zone of inhibition, while the AFs diffused from the antimicrobial disks into the surrounding YPD agar media and inhibited fungal cell growth in a surrounding ring. In the absence of added IL, no inhibition of *C. albicans* growth was seen for Fluconazole and Itraconazole, while only minimal inhibition was observed for both Clotrimazole and Ketoconazole (Figure 2A). With progressively increasing amounts of both [HMIM]Cl and [OMIM]Cl, ZOIs increased for all Afs, including Fluconazole and Itraconazole, which appear to be ineffective without IL in the Kirby–Bauer disk assay. Experiments were performed in triplicate and ZOIs were measured for all plates. The average ZOI was plotted with *p* < 0.05 confidence intervals, demonstrating statistically significant increases in ZOI for all four AFs and for both [HMIM]Cl or [OMIM]Cl (Figure 2B).

**Figure 3:**
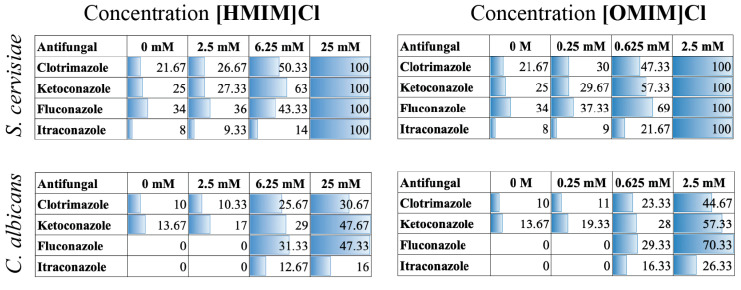
Kirby–Bauer disk diffusion assay diameters for four different AFs with two different ILs, using both *S. cerevisiae* and *C. albicans.* Diameters are measured in mm and represent the average of a single measurement on three replicate growth plates. A value of 100 indicates complete lethality as seen by no growth on plates.

**Figure A1:**
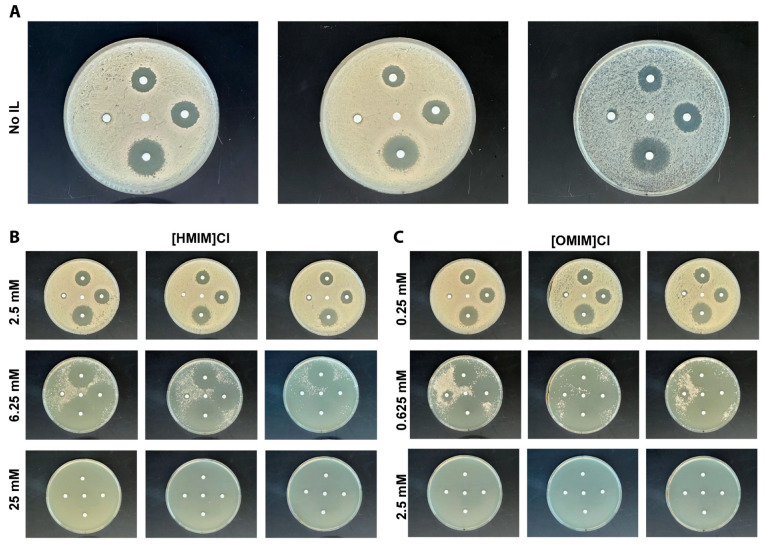
Kirby–Bauer disk diffusion assay with indicated concentrations of IL in YPD media plates with *S. cerevisiae*. Disks orientation from top in clockwise direction: Clotrimazole, Ketoconazole, Fluconazole, and Itraconazole. Assay performed in triplicate. (**A**) No Ionic Liquid added to plates. (**B**) [HMIM]Cl. (**C**) [OMIM]Cl.

Similar experiments testing the combinatorial effects of [HMIM]Cl or [OMIM]Cl were performed on *S. cerevisiae* using Kirby–Bauer disk assays and quantified for changes in ZOI (Appendix Figure A1). ZOI measurements (mm) from both *C. albicans* and *S. cerevisiae* were compiled, and the averages are shown numerically as well as with shading and plotted in Figure 3. *S. cerevisiae* was more sensitive to both [HMIM]Cl and [OMIM]Cl, as shown by the complete inability to grow on plates with 25 mM and 2.5 mM, respectively, and represented in Figure 3 as a complete lack of growth (value = 100). Furthermore, as seen in liquid cultures, *S. cerevisiae* was also sensitive to Fluconazole and Itraconazole without the addition of IL, suggesting greater susceptibility of *S. cerevisiae* to these AFs compared to *C. albicans*. Finally, at intermediate concentrations of both [HMIM]Cl and [OMIM]Cl, a combinatorial effect of IL + AF was evident for all four AFs in *S. cerevisiae,* similar to observations in *C. albicans*.

### 3.3. Combination of ILs and Antifungals Using Microbroth Dilutions in Checkerboard Assay Indicate Enhanced Fungicidal Activity

**Figure 4:**
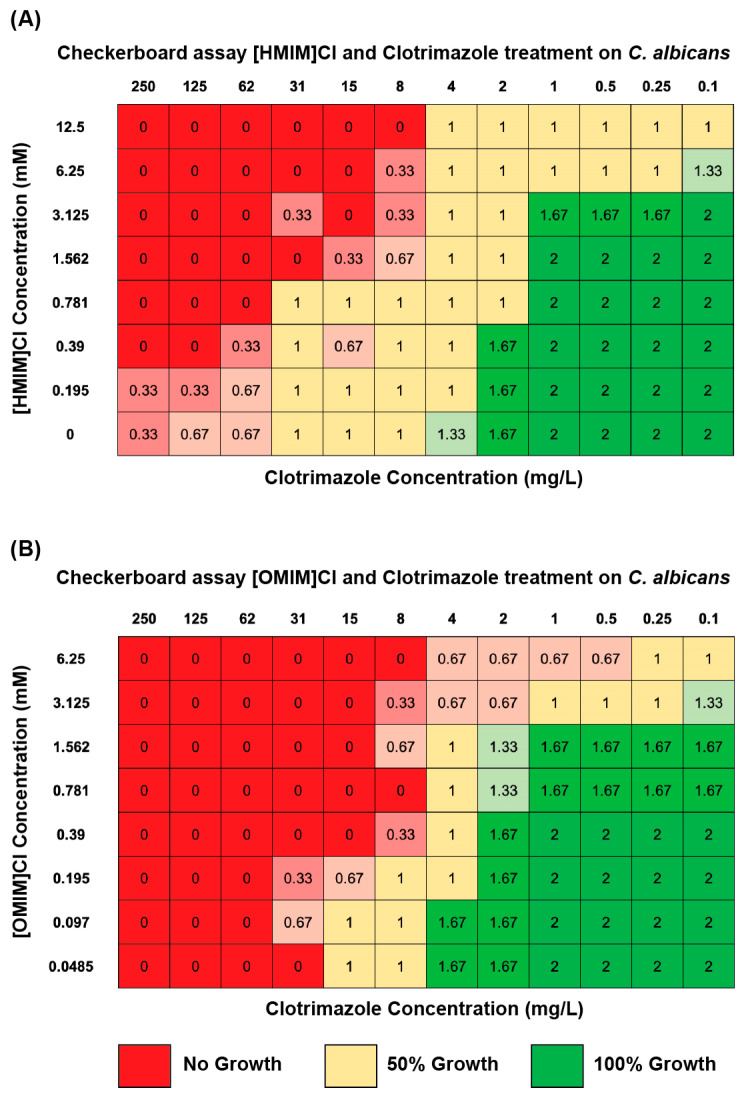
The antifungal Clotrimazole was combined with (**A**) [HMIM]Cl or (**B**) [OMIM]Cl in a 96-well plate with YPD to determine the combined effect of IL + antifungal to inhibit growth of *C. albicans*. After 24 h, liquid cultures were plated on YPD plates to evaluate growth. The final concentration of Clotrimazole (mg/L) decreases moving left to right and the concentration of the indicated ionic liquid (mM) decreases moving top to down. The numbers on the figure represent the average growth across the triplicates which were visually scored: 2 (green) indicates full uninhibited growth, 1 (yellow) indicates interrupted growth, and 0 (red) indicates no growth.

In order to evaluate if the same combinatorial effect was present in liquid culture and furthermore to identify the specific concentrations at which a combined effect may be present, checkerboard assays were performed. Clotrimazole was chosen as a representative azole antifungal in consideration that (1) all four AFs behaved in similar fashion in Kirby–Bauer disk assays and (2) Clotrimazole is the most frequently utilized topical antifungal for which ILs may be applicable. These assays were completed in triplicate for both [HMIM]Cl and [OMIM]Cl with the addition of Clotrimazole on *C. albicans*. In this assay, both IL and AF were loaded into a 96-well plate YPD liquid culture plate in double serial dilutions with decreasing IL from top to bottom and decreasing AF from left to right. Each well represents a different final concentration of both drugs in combination. Growth of the target microorganism was measured as [1] growth in liquid media by OD_600_ nm, representing fungistatic interactions and [2] growth on a YPD plate post-treatment as fungicidal (Figure 4).

Checkerboard assays were performed in 96-well plates with Clotrimazole + [HMIM]Cl or [OMIM]Cl on *C. albicans*. These culture plates were grown in an incubator shaker for 48 h at 23 °C, and thereafter *C. albicans* was mixed by pipetting and then spotted onto YPD solid plates and allowed to grow for an additional 48 h. After 48 h, plates were visually scored for no growth (0), 50% growth (1), or 100% growth relative to PBS control (2). Experiments were performed in triplicate, and scores were averaged per well. OD_600_ nm measurements from 96-well plate cultures prior to plating indicated similar patterning (Supplemental File S1) to plated yeast. Averaged results of the experiment indicate an antifungal effect of both [HMIM]Cl and [OMIM]Cl at the highest concentrations of 12.5 mM and 6.25 mM, respectively, although without the addition of Clotrimazole this effect was not completely fungicidal. Similarly, at the highest concentrations of Clotrimazole, 250 and 125 mg/L, *C. albicans* exhibited no growth. For both [HMIM]Cl and [OMIM]Cl, a combinatorial antimicrobial effect was evidenced at intermediate concentrations of both IL + AF which was not elicited when only a single drug was used. For example, without the addition of [HMIM]Cl treatment, *C. albicans* was able to demonstrate 50% growth post-treatment at 31 mg/L, while the addition of 1.562 mM [HMIM]Cl treatment decreased the amount of Clotrimazole required to demonstrate 50% growth to 4 mg/L. This trend scaled for multiple combinations of IL + AF for both ILs and demonstrates a characteristic synergistic interaction—diagonal shape of inhibited growth [34]. As seen in both the MIC50 and Kirby–Bauer disk assays, longer alkyl lengths exhibited a stronger overall antifungal effect at lower concentrations but appeared to elicit only a slightly stronger combinatorial effect. We measured this by calculating the fractional inhibitory concentration (FIC) score ((MIC50 IL + AF)/(MIC50 AF)) + ((MIC50 IL + AF)/(MIC50 IL)). In this well-established measure of combined interactions, a score < 0.5 indicated synergistic interaction, 0.5–4 potential combinatorial interaction or no effect, and >4 antagonistic interaction [30,35]. Based on data from checkerboard assays using [HMIM]Cl and [OMIM]Cl + Clotrimazole in *C. albicans*, we calculated FIC scores of 0.50 vs. 0.38, respectively. Finally, it is expected that these MIC50 values differ from those reported in Section 3.1 considering that these values represent concentrations at which these formulations are fungicidal in checkerboard growth assays and not just fungistatic as presented for microbroth dilution experiments in Figure 1.

In agreement with results from microbroth dilutions, fungicidal checkerboard assays with IL + AF indicate borderline synergistic and solidly additive interactions that are highly concentration dependent, suggesting that the synergistic effect is only present at specific concentrations of IL + AF. To further evaluate the mechanism behind the evidenced combined effects, candidate concentrations/combinations which elicited the strongest synergistic interactions, IL + AF concentrations, were chosen for further study in cell permeability assays.

[HMIM]Cl + Clotrimazole measured at 50% growth

(1.562 mM/6.25 mM) + (4 mg/L/15 mg/L) = 0.50

[OMIM]Cl + Clotrimazole measured at 50% growth

(0.39 mM/3.25 mM) + (4 mg/L/15 mg/L) = 0.38

### 3.4. Combinations of ILs + Clotrimazole Enhance Permeability of C. albicans in Liquid Culture

**Figure 5:**
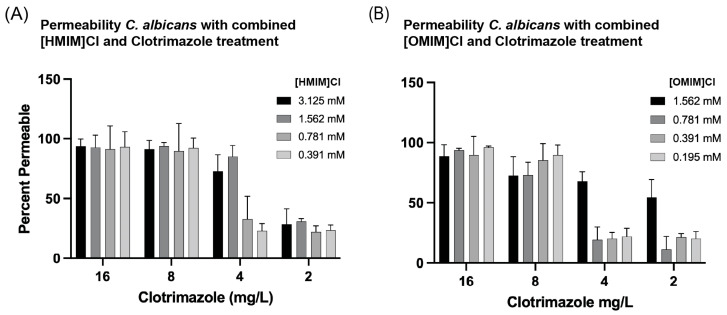
The antifungal Clotrimazole was combined with (**A**) [HMIM]Cl or (**B**) [OMIM]Clin liquid culture to determine the combined effect of IL + antifungal to permeabilize the *C. albicans*. After 24 h, Propidium iodide at a final concentration of 5 mg/mL was added to liquid cultures and fluorescence of cells at 595 nm was measured for each well. The percentage of permeable cells was determined as a ratio of PI-positive cells divided by total number of cells analyzed. Experiments were performed in triplicate and error bars represent confidence intervals *p* < 0.05.

The permeabilizing effects of 3-methylimidazolium chloride ILs, differing in alkyl chain length + Clotrimazole in *C. albicans* using flow cytometry were observed. While the membrane destabilizing effects of ILs have been thought to be the primary driver of enhancing antimicrobial properties in drug combinations, this study specifically tested this in the case of [HMIM]Cl and [OMIM]Cl to elucidate potential mechanisms of microbial inhibition induced by treatment with ILs. Flow cytometry was used to measure the microbial cell wall permeability in the presence of IL exposure through infiltration of propidium iodide into the cell after 24 h treatment at 23 °C. Propidium iodide, a DNA stain, will only enter cells in which the cell membrane is destabilized, and thus yeast cells exhibiting an increased fluorescent signal in the PE channel indicate membranes which have been permeabilized. Comparing the fluorescent signal intensities between our positive control, 3% cetrimonium bromide (CTAB), and our treatment of combined IL + AF, we were able to calculate the percentage of permeability. On a fundamental level, there is a direct correlation between greater bacterial cell permeability and longer alkyl chain lengths. When treated with IL alone, *C. albicans* becomes permeable at 12.5 mM [HMIM]Cl and 3.125 mM [OMIM]Cl within 2-fold of the respective MIC50 values. Using combinatorial treatments of IL + AF *C. albicans* exhibited increased permeability to PI stain after 24 h. In the case of [OMIM]Cl + Clotrimazole, combined treatment did not appear to specifically enhance permeability of *C. albicans* to PI (Figure 5B). In contrast, [HMIM]Cl + Clotrimazole improved entry of PI into C. albicans at 1.562 mM and 4 mg/L, respectively (Figure 5A). For [HMIM]Cl, this value is lower than the independently derived MIC50 values and fungicidal tests, while for Clotrimazole a value of 4 mg/L is greater than the observed MIC50 value of 1 mg/L and less than the 50% fungicidal value of 15–31 mg/L. Though not as pronounced as the Kirby–Bauer disk assay and checkerboard assays, results for [HMIM]Cl support that a combined IL + AF treatment leads to an increase in *C. albicans* permeability. It is important to note that while these experiments were performed for a shortened amount of time, 24 h vs. 48 h for MIC assays, it is impossible to disentangle whether the evidenced increased permeability is a primary effect from the treatments or a secondary effect from cell death.

### 3.5. Ionic Liquid and AF Cytotoxic Effects on Human Cells

**Figure 6:**
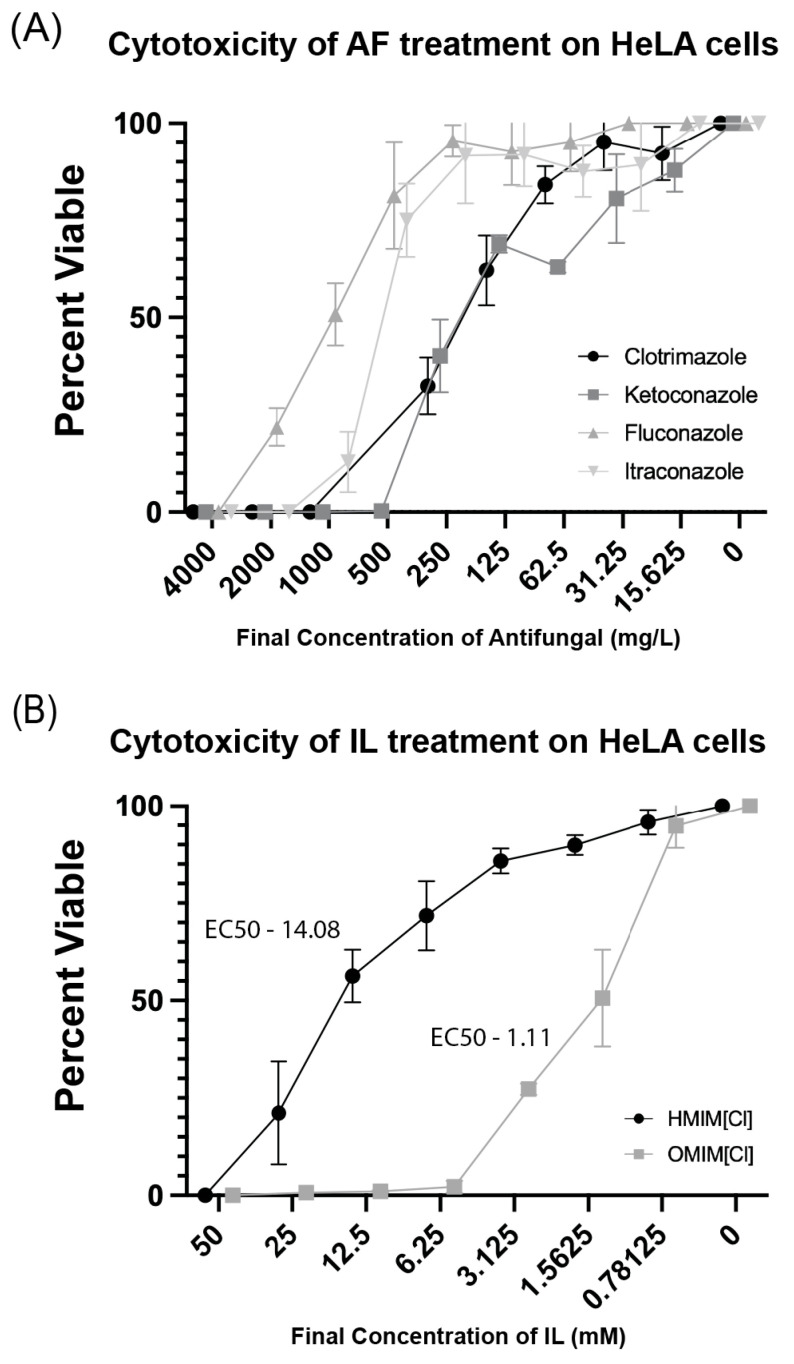
Cytotoxicity assays were performed for each of the (**A**) four antifungal compounds as well as (**B**) [OMIM]Cl or [HMIM]Cl in 96-well plates on HeLa cells. After 24 h of exposure to the compound, CellTiter-Blue was added and incubated for ~8 h and fluorescence was measured at 595 nm. Experiments were performed in triplicate and error bars represent confidence intervals *p* < 0.05.

To understand the degree to which imidazolium chloride-based ILs of varying chain length are toxic to human cells, we treated HeLa cells with [HMIM]Cl or [OMIM]Cl with each of the four azole AFs for 24 h and evaluated cytotoxicity. Following 24 h of exposure in liquid culture at defined serial dilutions in a 96-well plate format, cells were evaluated for viability using CellTiter-Blue assay in which living cells can metabolize resazurin into resorufin, a fluorescent reporter molecule detected by plate spectroscopy. Consistent with findings for bacteria and yeast microorganisms, high concentrations of imidazolium chloride-based ILs are toxic to HeLa cells (Figure 6B). Moreover, HeLa cells treated with ILs with longer alkyl chain lengths [OMIM]Cl are less viable than when treated with [HMIM]Cl at the same final concentration. EC50 calculations were performed for both [HMIM]Cl and [OMIM]Cl, and results suggested 50% viability at 14.08 mM and 1.11 mM, respectively. Notably, these are within 2-fold concentration to MIC50 values for all four yeast species, suggesting a lack of specificity for fungi vs. mammalian cells. With respect to all four AFs, the degree of specificity for these drugs to fungi vs. human cells was clear (Figure 6B). While MIC50 values for all four AFs ranged from 1 to 8 mg/L, in a human cell culture model, EC50 values ranged from 150 to 1250 mg/L.

At face value, low specificity might suggest limited potential for these ILs to be used in combinatorial therapy with these AF treatments. One open question is whether addition of either of these ILs at sub-cytotoxic concentrations can improve effectiveness of AFs in fungi vs. mammalian cells. To evaluate this, we performed checkerboard assays for both [HMIM]Cl and [OMIM]Cl in the presence of Clotrimazole and calculated FIC scores as described in *C. albicans* checkerboard assays. In checkerboard assays using [HMIM]Cl and [OMIM]Cl + Clotrimazole in HeLa cells we calculated FIC scores of 2.0 and 0.75, respectively. Based on conventions, this indicated that a synergistic interaction is not present in HeLa cells for IL +Afs; however, it is worth nothing that, while the FIC score for [HMIM]Cl clearly indicates a lack of combined drug interactions, the FIC score for [OMIM]Cl is much closer to a synergistic interaction, though not past the somewhat arbitrary threshold.

[HMIM]Cl + Clotrimazole

12.5 mM/12.5 mM + 250 mg/L/250 mg/L = 2.0

[OMIM]Cl + Clotrimazole

1.562 mM/3.125 mM + 62.5 mg/L/250 mg/L = 0.75

## 4. Discussion

The past 20 years have seen an explosion in research around the topic of using ILs as a novel class of antimicrobial agent. The ability to create ILs with a wide variety of different cations and anions as well as differing alkyl tail lengths has allowed the creation of a suite of tunable molecules with widely varying properties. Previous work from our laboratory focused on the antibiotic potential of ILs both independently and in combination with antibiotics. In those studies, we found that ILs enhance antibacterial efficacy in a synergistic fashion, which motivated research into whether the same phenomenon is true in fungal organisms which have a different cell membrane structure and for which it has been shown that drug delivery is a major barrier in developing effective antifungal compounds. In this study, we tested the antifungal effects of imidazolium-based ILs on four model fungal cell lines. Like work demonstrated previously, ILs in our study elicited antifungal effects which appeared to scale with alkyl tail length Figure 1 [25]. However, also as seen previously, these and other ILs demonstrate significant cytotoxicity towards human cell lines. Specifically, we found the EC50 cytotoxicity of [OMIM]Cl and [HMIM]Cl in HeLa cells is 1.11 mM and 14.08 mM, respectively, which is comparable to the MIC50 concentrations of 3.25 mM and 25 mM for *C. albicans*. While ILs have long been hailed to be green solvents, several reports, including ours, have identified that ILs on their own can be hazardous to human cells [24]. The cause of this effect may be related to different attributes of ILs, such as the selection of specific IL-cations, or changes in hydrophobicity of IL related to IL-chain length [36]. Used as a standalone treatment, imidazolium-based ILs possess limited potential as antifungal agents.

In consideration of the limited effectiveness of ILs as standalone antimicrobial agents, we therefore focused on whether these ILs can enhance existing antifungal drugs in combinatorial treatments. For this study, we focused on one class of commonly used antifungal drug, azoles, known to function by inhibiting the production of ergosterol which leads to the disruption of the cell membrane of the fungi, permeabilizing and therefore killing the fungal cell [5,6]. On their own, all four azoles inhibited the four tested fungi at similar published concentrations (CSLI) in MIC tests and showed inhibition of *C. albicans* growth for two of four azoles in Kirby–Bauer assays (Figure 1 and Figure 2). More importantly, a pronounced antimicrobial effect was observed in combination with either [OMIM]Cl and [HMIM]Cl, and across multiple assays including Kirby–Bauer and liquid broth microdilution experiments. For example, in Figure 2 there is strong evidence of a combinatorial effect, as regions of the agar plate with IL or AF only do not inhibit *C. albicans* growth as evident when both are in combination. Additionally, an increase in the permeability of *C. albicans* was observed when treated with a combination of [HMIM]Cl and Clotrimazole, though whether this effect is due to direct permeabilization of the membrane or PI entering due to cell death remains unknown. Finally, our checkerboard assays confirm the synergistic relationship of both [OMIM]Cl and [HMIM]Cl with Clotrimazole on *C. albicans* with FIC scores of 0.250 and 0.375, respectively. Notably, the enhancement of growth inhibition and cell permeabilization in *C. albicans* occurs at 1.563 mM for [HMIM]Cl, which in cytotoxicity assays on HeLa cells demonstrated low levels of toxicity (Figure 6B).

Taken together, these findings are highly comparable to our previous work with ILs + antibiotics, which may suggest that ILs may enhance drug delivery by generally disrupting the cellular membrane in both bacteria and fungi. This may also explain why ILs are toxic to human cells, although the exact molecular interactions between ILs and cell membranes have yet to be tested. Despite this, in both studies, while ILs are clearly cytotoxic to mammalian cells at high concentrations, at lower concentrations they are not toxic and yet still enhance antimicrobial efficacy, suggesting a potential for clinical application. Future studies will focus on developing IL + AF formulations for topical treatment of non-invasive mycoses, where ILs can significantly improve AF efficacy with only limited cytotoxicity to host cells/tissues. Moreover, this work is especially relevant at a time when studies have recently shown that multiple *C. albicans* strains have developed resistance to azole antifungals such as Clotrimazole [37,38]. Ultimately, results presented in this study were successful in showcasing the ability of ILs to increase the efficacy of AFs in general and Clotrimazole specifically in *C. albicans* at concentrations that are sub-cytotoxic to human cell lines.

## 5. Conclusions

The demonstration of increased antimicrobial efficacy with IL + AF combinations provides motivation for future combinatorial treatment experiments on additional fungal pathogens, with novel IL formulations, and using experimental methods that will address antimicrobial mechanisms. In the context of membranes, ILs are described and hypothesized as having “soft interactions” or “hard interactions”; respectively, (1) ILs can integrate into cell membranes, thereby altering phospholipid arrangements, fluidity, membrane potential, and viscoelasticity, thereby impacting processes which include protein diffusion, stability, and function which disrupt key cellular activities such as signaling, recognition, migration, and division, potentially leading to apoptosis, or (2) ILs can significantly impact cell membranes by altering permeability, nucleating pores, or even causing complete membrane disruption, and as a result disturbing biochemical gradients, leading to intracellular content leakage or extracellular material penetration, ultimately impairing membrane function and protein activity, often resulting in cell death. Given the wide range of tunable IL combinations, 10^18^, and multiple ways in which ILs may interact with the cell membrane to enhance AF efficacy, much work remains. Future studies should endeavor to elucidate the mechanistic interactions of ILs in combination with AFs on fungal and human membranes with the use of techniques such as super-resolution confocal microscopy and total internal reflection microscopy (TIRF), which can illuminate these molecular interactions.

## Data Availability

All data is available in the article or in the Supplemental Files supplied.

## Supplementary Materials

The following supporting information can be downloaded at: https://www.mdpi.com/article/10.3390/biom15121657/s1, Supplemental File S1: ILs Cytotoxic HeLa Checkerboard Assay.

## Abbreviations

The following abbreviations are used in this manuscript:

IL	Ionic Liquid
AF	Antifungal
[HMIM]Cl	1-hexyl-3-methylimidazolium chloride
[OMIM]Cl	1-octyl-3-methylimidazolium chloride
